# The Human OCTN Sub-Family: Gene and Protein Structure, Expression, and Regulation

**DOI:** 10.3390/ijms25168743

**Published:** 2024-08-10

**Authors:** Michele Galluccio, Martina Tripicchio, Lorena Pochini

**Affiliations:** 1 Laboratory of Biochemistry, Molecular Biotechnology, and Molecular Biology, Department of Biology, Ecology and Earth Sciences (DiBEST), University of Calabria, Via P. Bucci 4c, 87036 Arcavacata di Rende, Italy; tripicchio.martina9@gmail.com; 2 Institute of Biomembranes, Bioenergetics and Molecular Biotechnology (IBIOM), National Research Council (CNR), Via Amendola 122/O, 70126 Bari, Italy

**Keywords:** OCTN1, OCTN2, gene expression and regulation, SLC22 family, IBD, carnitine

## Abstract

OCTN1 and OCTN2 are membrane transport proteins encoded by the *SLC22A4* and *SLC22A5* genes, respectively. Even though several transcripts have been predicted by bioinformatics for both genes, only one functional protein isoform has been described for each of them. Both proteins are ubiquitous, and depending on the physiopathological state of the cell, their expression is regulated by well-known transcription factors, although some aspects have been neglected. A plethora of missense variants with uncertain clinical significance are reported both in the dbSNP and the Catalogue of Somatic Mutations in Cancer (COSMIC) databases for both genes. Due to their involvement in human pathologies, such as inflammatory-based diseases (OCTN1/2), systemic primary carnitine deficiency (OCTN2), and drug disposition, it would be interesting to predict the impact of variants on human health from the perspective of precision medicine. Although the lack of a 3D structure for these two transport proteins hampers any speculation on the consequences of the polymorphisms, the already available 3D structures for other members of the SLC22 family may provide powerful tools to perform structure/function studies on WT and mutant proteins.

## 1. Introduction

The solute carrier superfamily (SLC) includes more than 450 proteins clustered in 66 families and comprises about 9% of the human membrane proteome [1,2]. These proteins play a crucial role in human cell metabolism, allowing, on the one hand, the tightly regulated absorption and distribution of several substrates, such as amino acids, sugars, peptides, nucleosides, vitamins, ions, neurotransmitters, and drugs; on the other hand, they allow the elimination of catabolites and waste products. With its twenty-eight members, the SLC22 family represents one of the largest clusters of membrane transporters with broad specificity, including anions (OATs), cations (OCTs), both cation and zwitterion transporters (OCTNs) [3], and four still orphan members [1]. Very recently, an advance in knowledge of the substrate specificity and transport mechanism of some members of the SLC22 transporter family has been achieved through the solution of the 3D structure of *SLC22A1*, *SLC22A2*, and *SLC22A3* [4,5,6,7] and the organic anion transporter *SLC22A6* [8]. Three-dimensional structures of the members of the OCTN subfamily are not yet available. These two human transporters are of great interest for their involvement in physiopathology [9]. In rodents, the OCTN subfamily includes a third member, OCTN3, encoded by the *SLC22A21* gene, which is highly expressed in the testis, where it is involved in sperm maturation [10]. In humans, only two members, OCTN1 and OCTN2, encoded by the *SLC22A4* and *SLC22A5* genes, respectively, have been described [11,12]. The interest of the scientific community in this transporter’s subfamily resides both in its role in the drug ADME [10,13] and its involvement in human pathologies [14,15]. Indeed, both *OCTN1* and *OCTN2* genes are directly or indirectly linked with inflammatory diseases and cancer [14,16,17,18,19], and *OCTN2* may be considered a “gene-disease” since several single nucleotide polymorphisms (SNPs) and mutations of this gene are causative of systemic primary carnitine deficiency (CDSP). It is a rare autosomal recessive disease that is characterized by a very low intracellular level of carnitine due to a strong decrease or eradication of carnitine uptake, which causes impaired fatty acid oxidation in skeletal and heart muscles, leading untreated patients to experience issues like encephalopathy, cardiomyopathy, liver problems, coma, heart failure, and sudden unexpected death [20,21,22,23,24].

The purpose of the manuscript is to provide the readers with current knowledge on *OCTN* gene/protein expression, structure, polymorphisms, mutations, and regulation. Moreover, the critical gaps in knowledge concerning the various configurations of these proteins that may affect function will be discussed.

## 2. *SLC22A4/OCTN1* Gene and Protein

**Figure 1:**
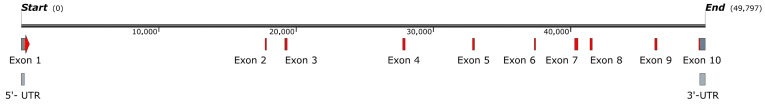
*SLC22A4* gene map. The exons and UTRs are indicated as red and grey squares, respectively. The size of each square is proportional to its length.

The *OCTN1* gene is located at chromosome 5 (5q31.1) in the inflammatory bowel disease 5 (IBD5) locus, known for its implication of susceptibility to Crohn’s disease (CD), ulcerative colitis (UC), and rheumatoid arthritis (RA) [14,25,26,27,28]. The *SLC22A4* gene counts 49,797 nucleotides and 10 exons (Figure 1). The exons 1 and 10 include 5′-UTR and 3′-UTR, respectively.

### 2.1. OCTN1 Gene Variants (SNPs)

When searching for gene variants on the dbSNP database (https://www.ncbi.nlm.nih.gov/snp/?term=slc22a4, accessed on 20 May 2024), 17,834 sequence variants were found, most of which were not defined as pathogenic. Among these, rs1050152 is the most commonly cited SNP associated with Crohn’s disease [14,27,29]. The consequences of the resulting L503F substitution have been verified by the in vitro experimental system of proteoliposomes [29] (see Section 2.2). The rs768484124 polymorphism causing a G>A, T substitution in the 5′-UTR region is the only one considered likely to be pathogenic, while the intronic variant rs3792876 (NC_000005.10:132301615:C:T) has been classified as a risk factor for Crohn’s disease [30] and strongly associated with rs1050152 in type I diabetes among Spanish patients [31]. The link between *OCTN1* polymorphisms and inflammation-related pathologies has been extensively reviewed [14,32,33]. Recently, an untargeted metabolic phenotyping study was performed on 1191 serum samples from older individuals (between 56 and 84 years old) using liquid and gas chromatography-mass spectrometry metabolomics stratified across a frailty index (FI) [34]. The analysis identified 12 significant metabolites, 6 of which were carnitines that differentiate frail from non-frail phenotypes, highlighting the association of the intronic *OCTN1* polymorphism rs419291 with high carnitine levels and healthy ageing [34]. More than 93% of the *OCTN1* SNPs reported in the dbSNP database are intronic, and 510 polymorphisms are classified as missense. Among these, 444 involve exons of the canonical transcript isoform. In particular, 88 codons have 2 different amino acid variants, 9 codons have 3 amino acid variants, and the Asp 138 codon has been found mutated in Glu, Asn, Gly, and Val. The functional consequences of these missense variants are defined as uncertain. One possible way to predict the outcomes is to analyze their proximity to the putative substrate binding sites.

### 2.2. OCTN1 Transport Mechanisms

**Figure 2:**
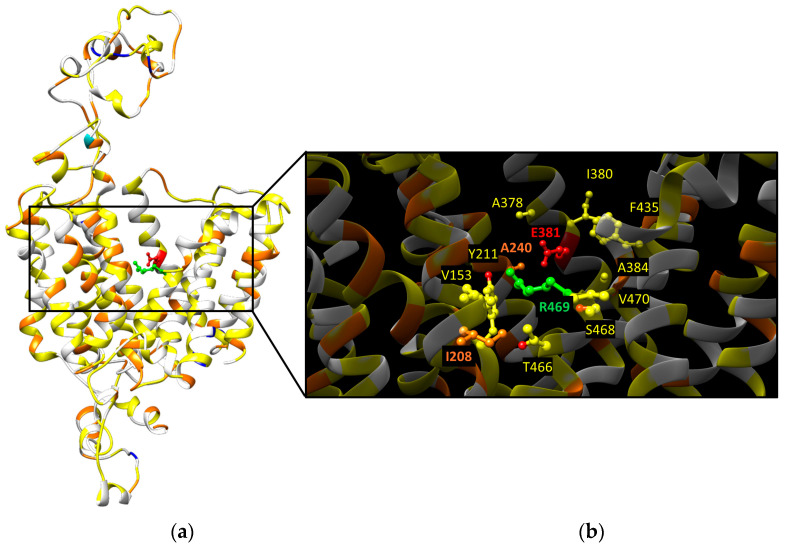
Distribution of polymorphisms on the SLC22A4 homology model obtained as described in [9]. (**a**) The positions not affected by polymorphisms are indicated as white ribbons. The amino acids, which have been found mutated in one, two, three, or four other different amino acids, are indicated in yellow, orange, blue, and cyan, respectively. The two target amino acids of the organic cation and zwitterion binding sites are indicated in red and green, respectively. (**b**) Zoom in on the putative substrate binding sites with the amino acids within 4 Å colored as in (**a**).

Experimental data would suggest the existence of two different pathways and/or binding sites on the OCTN1 transporter, each involved in the recognition of specific substrates (organic cations or zwitterions) [9]. The E381 residue, corresponding to E386, E387, and E390 of OCT1, OCT2, and OCT3, respectively, already identified as crucial for the substrate translocation pathways [4,5,6,7], has been identified by our group as the putative Na^+^ binding site by molecular dynamic simulation [9]. Interestingly, docking experiments with prototypical OCTN1 substrates, such as carnitine and TEA, performed in the presence or absence of sodium, showed a different behavior; in the presence of sodium, E381 was not accessible to TEA, unlike carnitine, which interacted with R469 [9]. The proteoliposome system has been exploited to test the effect of L503F substitution on the transport activity. It has been found to reduce the Vmax without affecting the Km. This would suggest that the substrate binding site is not modified, whereas the conformational changes necessary for acetylcholine efflux are impaired [29]. The homology model built using human OCT3 as a template has been employed to analyze the position of the *OCTN1* missense variants with respect to the two putative binding sites (Figure 2). In the neighborhood within 4 Å of the zwitterion binding site represented by R469, 7 variants that might affect OCTN1 activity have been highlighted (Figure 2b). Among these, the Y211C substitution could have the strongest effect due to the side chain modification. The same analysis performed around the organic cation binding site represented by E381 highlighted six variants, among which A240V might potentially hamper the substrate binding due to the increased steric hindrance of the valine side chain with respect to the alanine one (Figure 2b).

### 2.3. OCTN1 Somatic Mutations 

**Figure 3:**
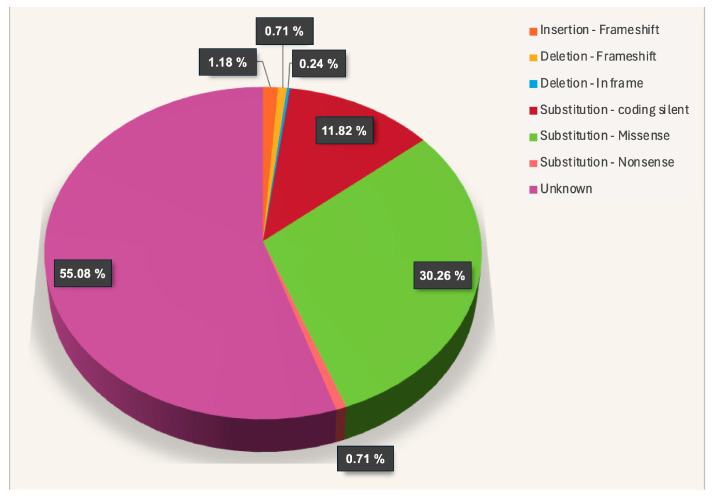
Somatic mutations of the *OCTN1* gene in cancer.

The increase in whole-genome sequencing projects has revealed an increment of the described mutations for several genes. Indeed, two intergenic and two intronic mutations have been described for the *OCTN1* gene in radiation-induced sarcoma [35]. The role of OCTN1 in cancer has been reported not only as an anticancer drug transporter but also in the induction of epithelial-mesenchymal transition (EMT), migration, and the invasion of human lung cancer cells [33]. Looking at the catalogue of somatic mutations in cancer (COSMIC, https://cancer.sanger.ac.uk/cosmic/gene/analysis?ln=SLC22A4#distribution, accessed on 26 June 2024), 423 mutations of the *OCTN1* gene have been reported. Among these, 12% are silent, and the number of frameshift mutations with the possible worst effects is under 2% (Figure 3).

**Figure 4:**
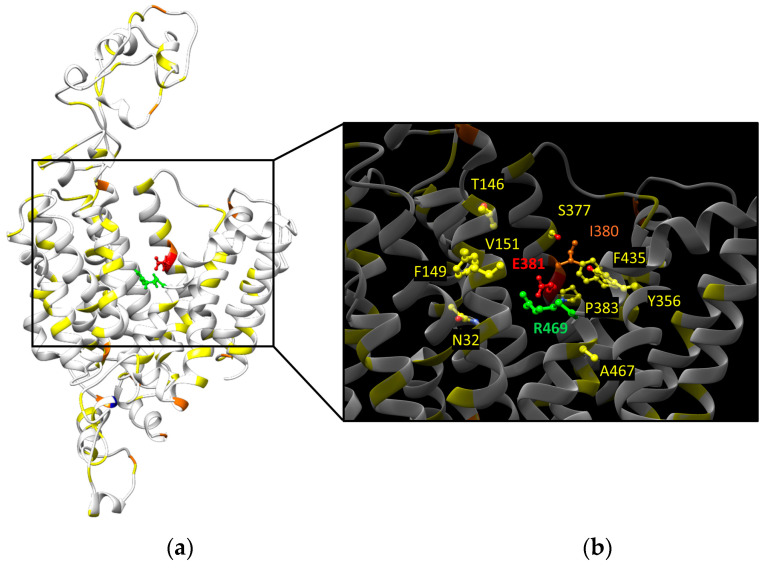
Distribution of somatic mutations in cancer on the SLC22A4 homology model obtained as described in [9]. (**a**)The amino acids that have been found mutated in one, two, or three different other amino acids are indicated in yellow, orange, and blue, respectively. The two target amino acids of the organic cation and zwitterion putative binding sites are indicated in red and green, respectively. (**b**) Zoom in on the substrate binding sites with the amino acids within 4 Å colored as in (**a**).

Structure/function studies are needed to unveil the consequences deriving from the missense mutations that count for 30% of the total. A possible strategy to predict the functional consequences of these mutations is to check their proximity to the putative substrate binding sites, as described in Figure 2. The 128 missense mutations have been highlighted in the OCTN1 homology model (Figure 4a).

From this “in silico” analysis, less than 10% of the missense mutations in cancer are in the proximity of the substrate binding sites: five mutations might affect the zwitterion binding site, while six mutations might disturb the organic cations translocation pathway (Figure 4b). This would suggest a correlation between OCTN1 and cancer related to a potential modulation of the transport activity.

### 2.4. OCTN1 Promoter/Enhancer 

The promoter region of the *SLC22A4* gene has been extensively studied due to its involvement in inflammatory bowel diseases (IBDs) and drug transport. In 2007, for the first time, Maeda et al. investigated OCTN1 regulation by the rheumatoid arthritis-associated transcriptional factor RUNX1 and inflammatory cytokines [36]. In 2009, Tahara et al., starting from HepG2 genomic DNA, amplified and cloned about 400 bp of the *OCTN1* promoter region for a luciferase assay [37]. Even though six new variants have been identified, none of them have caused modulation of luciferase activity. Some years later, the occurrence of four *OCTN1* promoter variants, rs3761661, rs3761660, rs162887, and rs460271, in patients with CD and in controls were examined, and it was concluded that some *OCTN1* functional promoter haplotypes could affect the clinical phenotype of CD in Koreans, represented by a predisposing factor for the development of penetrating behavior characterized by intestinal perforation, inflammatory mass, and/or abscess [38]. A well-known *OCTN1* polymorphism, rs1050152-CT, clearly associated with inflammatory bowel diseases [29], has been associated with a major molecular response (MMR) to imatinib, which is the first-line drug used for the treatment of patients affected by chronic myeloid leukemia (CML) [39]. Thanks to high-throughput NGS studies in patients with CML, two novel polymorphisms, rs460089 and rs2631365, have been described as *OCTN1* and *OCTN2* promoters, respectively. In particular, rs460089 and rs2631365 were in highly significant linkage disequilibrium with several regulatory loci in the introns of *SLC22A4* and *SLC22A5*. Interestingly, the heterozygous (G/C) genotype, rs460089-GC, was positively associated with the maintenance of treatment-free remission (TFR) in patients from the European Stop Kinase Inhibitor (EURO-SKI) trial [40].

**Figure 5:**
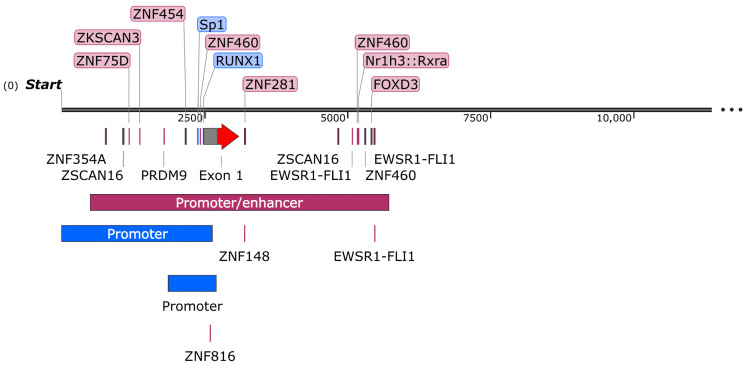
*OCTN1* promoter/enhancer scheme. The experimentally proven and bioinformatically predicted features are indicated in blue and purple, respectively. The corresponding regions are described in the text. The predicted transcription factors of Table 1 are also shown.

The *OCTN1* promoter/enhancer region deposited in the GeneHancer database counts 5218 bp [41]. Following a bioinformatics analysis with the JASPAR CORE 2024 database [42], several transcription-factor binding sites with a score higher than 600 have been predicted, most of which belong to the zinc finger protein family (Table 1), highlighting several putative regulation pathways for the *OCTN1* gene (Figure 5).

### 2.5. OCTN1 Gene and Protein Expression and Regulation 

The *OCTN1* gene is ubiquitously expressed and was identified and cloned for the first time in 1997 [13,43]. The mature mRNA (ENST00000200652.4) counts 2234 nt and codes for a protein of 551 amino acids. No additional protein isoforms are reported in any database, although additional transcripts have been predicted by bioinformatics and reported in the Ensembl and the NCBI/gene databases (Table 2).

The expression of the *OCTN1* gene could be regulated by the lncRNA MIR3936HG. This lncRNA, transcribed in the opposite direction with respect to the *OCTN* genes, is characterized by 8 exons and 1802 nt, and it overlaps with part of the *OCTN1* and the 5′-UTR of the *OCTN2* gene. It would be interesting to know if this lncRNA is ubiquitously expressed as the *OCTN1* gene or if its expression is cell type-specific or changes depending on the physiological state of the cell, as demonstrated for another antisense RNA (SLC16A1-AS1) in many types of cancer [44]. It would also be interesting to know if the expression of this antisense RNA negatively regulates the expression of the *OCTN1* gene, as is already seen for another member of the SLC superfamily [45].

**Figure 6:**
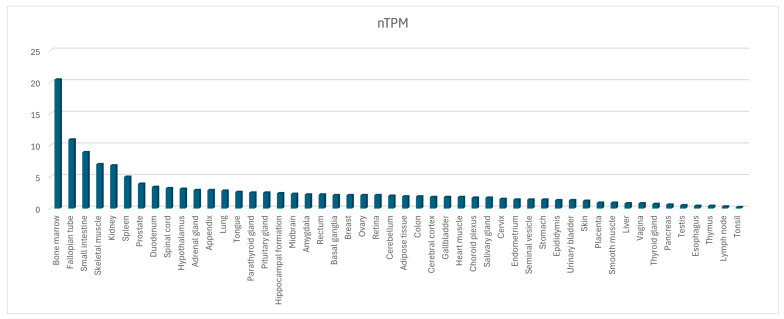
Tissue expression profile of the *SLC22A4* RNA; nTPM, normalized transcript per million, adapted from https://www.proteinatlas.org/ENSG00000197208-SLC22A4/tissue#rna_expression (accessed on 20 June 2024).

An RNA seq analysis from the Human Protein Atlas (HPA) and Genotype-Tissue Expression (GTEx) projects has highlighted the ubiquitous expression of this gene even when it has some differences in tissue expression. The amount of RNA, measured as transcript per million and retrieved from the different data sources, was normalized separately using the trimmed mean of M-values (TMM). The resulting normalized transcript expression values (nTPM) were calculated for the gene in every sample (Figure 6).

OCTN1 is highly expressed in marrow. Considering its expression in both immature and mature erythrocytes, it has been speculated that it catalyzes the transport of compounds involved in erythroid differentiation, maturation, and growth [46].

## 3. *SLC22A5/OCTN2* Gene and Protein

**Figure 7:**
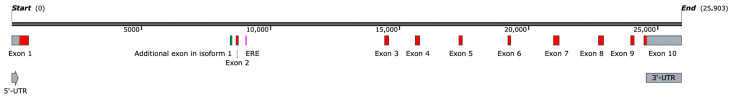
*SLC22A5* gene map. The exons and UTRs are indicated as red and grey squares, respectively. The size of each square is proportional to its real length. The additional exon of the *OCTN2* isoform 1 is shown in green. The size of each square is proportional to its length. ERE: estrogen-responsive element.

Although the genomic location in the IBD5 locus at chromosome 5 (5q31.1) certifies its involvement in inflammatory bowel disease [27,28], *OCTN2* may be considered as a “disease-gene”. Indeed, a plethora of *OCTN2* mutations are responsible for primary systemic carnitine deficiency (CDSP), classified in the Online Mendelian inheritance in Man (OMIM) database (https://www.omim.org/entry/212140) (accessed on 21 June 2024) [47,48]. *OCTN2* was cloned in 1998 [49]. The gene counts 25,903 nt coding for 2 isoforms. Isoform 2 is the canonical one, characterized by 10 exons (Figure 7), and it encodes a 557 amino acid protein with plasma membrane localization. An alternative splicing event may lead to the inclusion of an additional 72 bp from intron 1 (Figure 7). The resulting mRNA with 11 exons encodes a 581 poorly N-glycosylated inactive protein named OCTN2-VT, which is retained in the endoplasmic reticulum with a role that is unidentified at present [50].

### 3.1. OCTN2 Gene Variants (SNPs)

There are 10,171 variants for the *SLC22A5* gene reported in the dbSNP database (https://www.ncbi.nlm.nih.gov/snp/?term=slc22a5, accessed on 30 May 2024). Among these, 116 are classified as pathogenic, 71 are likely pathogenic, and 128 are benign. About 86% of these variants are intronic, and 652 are classified as missense. By exploiting the variant viewer tool of the UniProt database (https://www.uniprot.org/uniprotkb/O76082/variant-viewer, accessed on 31 May 2024), 162 pathogenic variants can be found associated with renal carnitine transport defects. Among these, rs377767449 is linked with a congenital myasthenic syndrome, while 76 are classified as causing primary systemic carnitine deficiency (CDSP) and, in most cases, the complete loss of carnitine transport.

### 3.2. OCTN2 Transport Mechanism

OCTN2 has been identified as a Na^+^-dependent high-affinity carnitine transporter [49]. Apart from carnitine, it can also transport organic cations in a sodium-independent way [49]. The existence of two pockets in the binding site specific to the carboxyl group and the ammonium ion has been hypothesized [51]. Recently, a machine learning-based prediction method for an *OCTN2* variant has been developed to predict functional consequences and helping in the diagnosis and treatment of CDSP [48]. In particular, 150 missense variants have been selected, spanning the entire secondary structure of OCTN2. All these variants have been expressed in HEK293T cells, and ^14^C-carnitine uptake has been measured. Seventy-one percent (one hundred and seven variants) showed a significant decrease in carnitine transport. For 37 variants, 2 of which were novel (V216L, G411V), the transport activity was less than 20% with respect to the WT [48].

**Figure 8:**
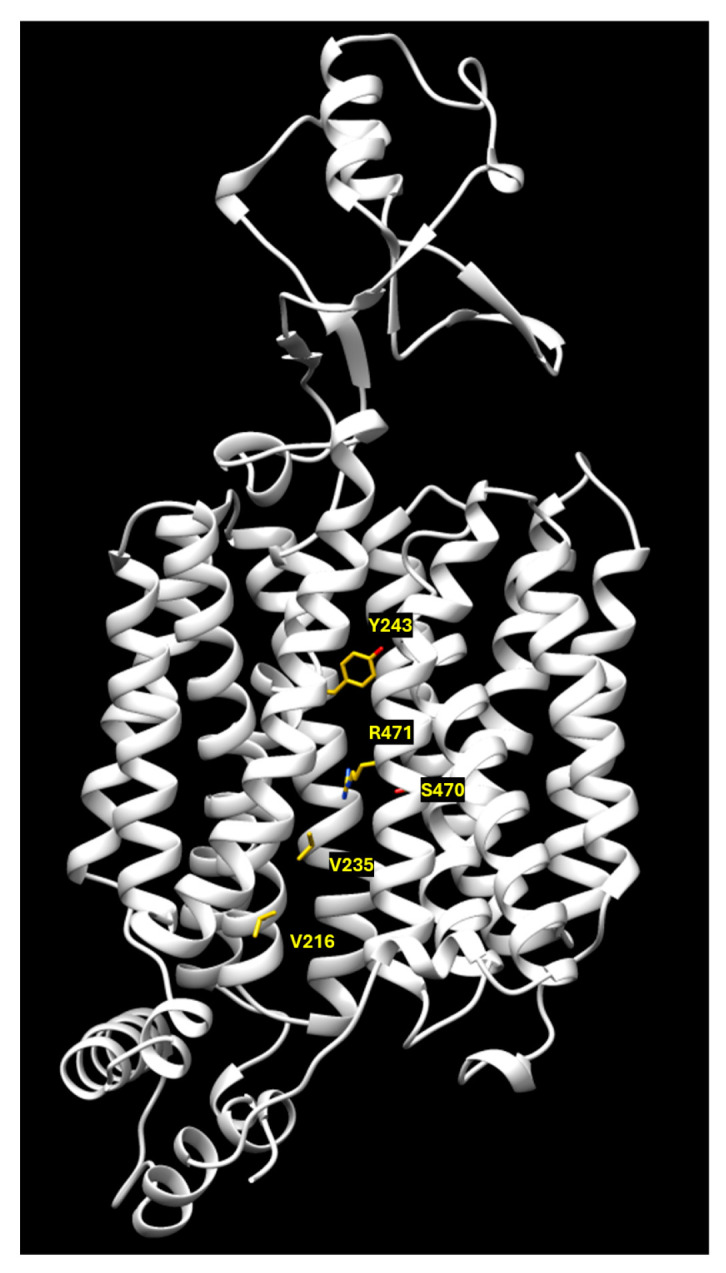
Ribbon representation of the 1–523 aa of the OCTN2 homology model retrieved from AlphaFold database. The loss of function variants protruding into the translocation pore are highlighted as yellow licorices.

Interestingly, the majority of loss-of-function variants (26/37) are located in transmembrane domains. The sub-cellular localization of the variants has also been investigated by exploiting a GFP-tagging strategy. Fifty-seven variants showed membrane localization, thirty-six variants displayed intracellular retention, and fifty-seven variants had mixed localization. Even though the function of the variants was strictly related to their membrane localization, some of them (p.V216L, p.V235G, p.Y243S, p.S470F, and p.R471C) were inactive despite the proper membrane localization. The loss of function was probably related to their position in the carnitine translocation pore, where the modification of amino acid side chains may hamper the substrate translocation pathway (Figure 8) [48].

### 3.3. OCTN2 Somatic Mutations 

**Figure 9:**
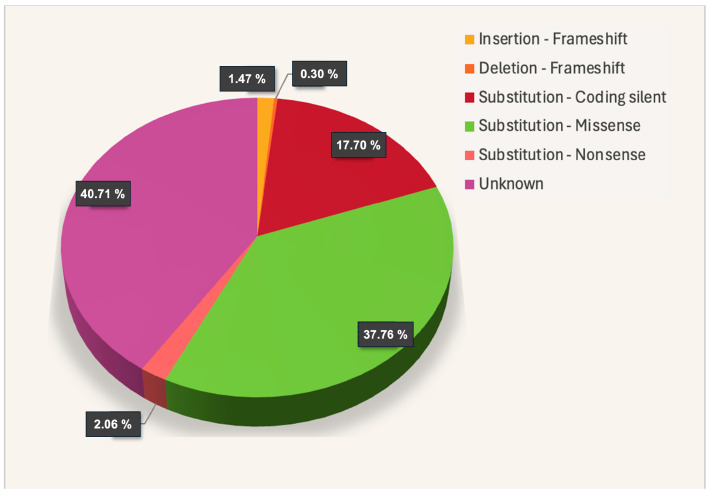
Somatic mutations of the *OCTN2* gene in cancer.

In the COSMIC database (https://cancer.sanger.ac.uk/cosmic/gene/analysis?ln=SLC22A5_ENST00000245407#distribution, accessed on 29 June 2024), 339 somatic mutations in cancer have been described for *OCTN2*, among which about 1% are represented by frameshift and 2% are nonsense mutations (Figure 9).

The application of the prediction model developed by Koleske et al. [48] would help in hypothesizing the functional consequences of the unknown or the missense mutations that represent 41% and 38% of the total, respectively.

### 3.4. OCTN2 Promoter/Enhancer

The −207G>C transversion in the *OCTN2* promoter (rs2631367) has been found in strong linkage disequilibrium with the 1672C→T polymorphism in the *OCTN1* gene, creating a two-allele risk haplotype (TC) enriched in patients affected by CD [27]. Moreover, this substitution results in the disruption of a heat shock transcription factor (HSF)-binding element (HSE) within the *OCTN2* promoter with a strong reduction of its transcriptional activity in heat-shocked cells [27]. The same transversion has been found associated with increased transcription levels in lymphoblastoid cell lines [37]. Although no direct evidence has been provided, a putative role in the disposition of several drugs, such as β-lactams [52] or carnitine derivatives [53], has been suggested. In a Japanese study, 94 patients with CD, 94 with UC, and 257 healthy controls were genotyped to test individual drug responsiveness to steroid drugs. Interestingly, the haplotype analysis between rs4646298 and rs2631368 in the *SLC22A5* promoter showed that the CG allele seemed to be a risk factor for steroid resistance [54]. The presence of an enhancer located 6 kb upstream of the *OCTN2* promoter has been predicted by a computational approach and tested by luciferase assay, demonstrating that this short sequence is necessary for the full activation of the *OCTN2* promoter. Indeed, the deletion of this enhancer region caused a 2.5-fold reduction in reported activity [55]. To investigate whether the different OCTN2 expression in different cancer cell lines is related to the methylation state of its promoter, the *OCTN2* genomic sequence was divided into three regions containing different CpG islands that were amplified and cloned into a luciferase reporter plasmid [56]. Among the three regions, only the one spanning −354 to +85 bp caused a strong increase (about a hundredfold) of the luciferase activity, showing an essential role in promoter activity. Moreover, the hypermethylation of this region caused an inhibition of the promoter activity in LS174T and HepG2 cells, and the DNA methylation degree was inversely correlated with the expression of OCTN2 in these cancer cells [56]. Given the role of the OCTN2 transporter in the uptake of the anticancer drug oxaliplatin, pretreatment with the demethylating agent decitabine may trigger an increase in OCTN2 expression, which improves the drug uptake. Taken together, these observations suggest that the use of demethylating reagents is a possible strategy for the optimization of pharmacotherapy that treats cancer. 

**Figure 10:**
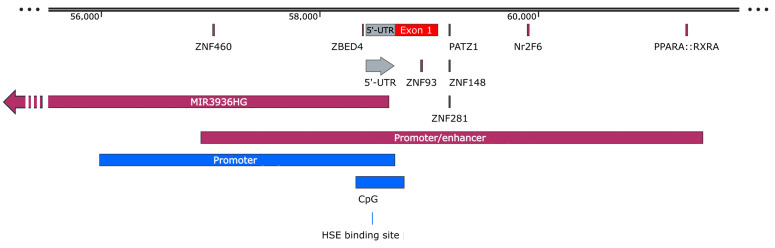
*OCTN2* promoter/enhancer scheme. The experimentally proven and the bioinformatically predicted features are indicated in blue and purple, respectively. The corresponding regions are described in the text. The predicted transcription factors of Table 3 and the lncRNA MIR3936HG are also shown.

The *OCTN2* promoter/enhancer region deposited in the GeneHancer database counts 4587 bp [41]. In the search for transcription factors with a score higher than 600 using the JASPAR CORE 2024 tool [42], in addition to the well-known OCTN2 regulator, peroxisome proliferator-activated receptor (PPAR)-α, other transcription factors have been predicted, with a prevalence of zinc finger proteins that could be involved in *OCTN2* regulation (Table 3 and Figure 10).

### 3.5. OCTN2 Gene and Protein Expression and Regulation 

*OCTN2* encodes two mature mRNAs. Isoform 2 is considered canonical, and it codes for a protein of 557 amino acids. Other additional protein isoforms have been predicted by bioinformatics and reported both in the Ensembl and the NCBI/gene databases (Table 4).

The stability of the *OCTN2* mRNA may be strongly influenced by the interaction between the MIR3936HG and the 5′-UTR of the *OCTN2* gene (see above). Indeed, it has been observed that lncRNAs may recruit RNA binding proteins to the 5′-UTR of a gene to protect it against possible nuclease targeting [57]. Moreover, in vitro, antisense oligonucleotides (ASOs) have been seen to block 5′-UTR elements affecting protein expression through increased ribosome occupancy [58].

**Figure 11:**
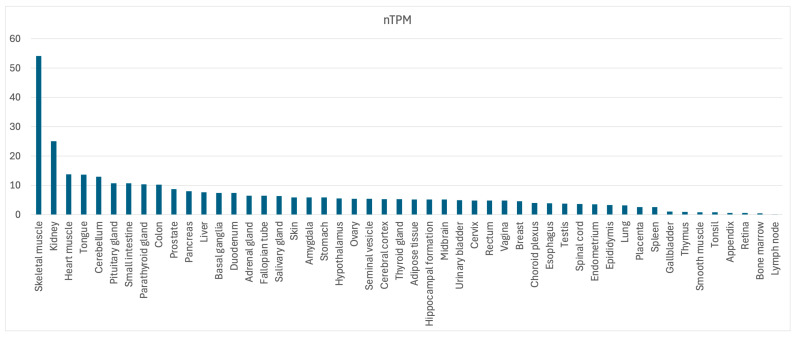
Tissue expression profile of the *SLC22A5* RNA. nTPM, normalized transcript per million, adapted from https://www.proteinatlas.org/ENSG00000197375-SLC22A5/tissue#rna_expression (accessed on 20 June 2024).

*OCTN2* is ubiquitously expressed, with particular abundance in skeletal muscle, kidney, intestine, heart, and brain (Figure 11) [32,59].

A down-regulation of the *OCTN2* mRNA has been observed in liver biopsies of patients treated with the antiepileptic drug carbamazepine (CBZ) [60]. Clofibrate, a lipid-lowering agent, has been seen as responsible for an increase in carnitine concentration in rat liver, thanks to the activation of PPARα [61,62], a transcription factor belonging to the nuclear hormone receptor superfamily [63]. The same increase in *OCTN2* mRNA concentration has also been confirmed in rat small intestine [64]. Moreover, the use of a PPARα agonist had the same effect in mice liver [65]. Even though humans and pigs have a 10-fold lower expression of PPARα, it has been demonstrated that the use of clofibrate triggers the up-regulation of *OCTN2* in pig liver, muscle, and enterocytes via PPARα [66]. All these findings suggest that *OCTN2* expression regulation may depend on the tissue-specific PPARα expression [66]. Since, in the human colon, PPARγ is more abundant compared to PPARα, it is reasonable that *OCTN2* expression may be primarily regulated by PPARγ. Indeed, the use of the PPARγ inducers thiazolidinediones (troglitazone and rosiglitazone TZDs) causes a strong increase in *OCTN2* mRNA expression quantified by RT-PCR in human colonocytes [67]. OCTN2 is expressed in the syncytiotrophoblasts of the human placenta [68], and it is up-regulated following forskolin-induced syncytialization [69]. However, under hypoxic conditions, its mRNA and protein levels, as well as PPARα, were reduced in human placental explants and BeWo cells by HIF1α [70]. The reduction of OCTN2 levels caused by hypoxia provides a possible explanation for the decrease in placental carnitine transfer seen in preeclampsia, leading to higher carnitine levels on the maternal side [70]. During fasting or energy restriction, PPARα activation by non-esterified fatty acids released from adipose tissues triggers an up-regulation of a set of genes involved in mitochondrial β/oxidation, among which is OCTN2. Thus, the increased OCTN2-mediated uptake of carnitine pushes mitochondrial fatty acid catabolism, minimizing the use of carbohydrates and proteins as fuels for mammals’ survival under energy deprivation conditions [71].

In patient-derived primary glioblastoma samples, an up-regulation of OCTN2 has been detected [72], which is a negative prognostic marker for survival. Moreover, a drug-mediated OCTN2 inhibition may slow down glioblastoma growth in a mouse model [72] and with high-grade serous epithelial ovarian cancer [73]. Conversely, it was shown to be down-regulated in virus- and nonvirus-mediated epithelial cancers, probably via promoter methylation [74]. OCTN2 is expressed in several breast cancer cell lines, and it is significantly up-regulated in estrogen receptor (ER)-positive cells [75]. Due to this positive correlation, the presence of estrogen-responsive elements (EREs) in the promoter region has been investigated. Interestingly, in the intron 2 region, a new ERE (GGTCA-CTG-TGACT) (Figure 6) has been found, demonstrating that *OCTN2* expression is regulated by estrogen and that OCTN2 is required for carnitine intake, lipid metabolism, and proliferation of breast cancer cells [75]. Recently, the activity and cell surface expression of OCTN2 in breast cancer cells has been investigated [76]. Carnitine transport has been positively correlated with the level of OCTN2 phosphorylated by AKT on threonine residues [76]. Thus, the use of AKT inhibitors may reduce carnitine transport, triggering a reduction in fatty acid oxidation and leading to reduced viability and increased apoptosis of cancer cells [76]. The muscle toxicity of levatinib, an oral tyrosine kinase inhibitor, may be a consequence of OCTN2 inhibition and carnitine decrease [77]. On one hand, OCTN2 may represent an alternative source of energy for cancer cells, and on the other hand, it can be used as an anti-cancer drug transporter, as with drug-carnitine conjugates [78].

## 4. Conclusions

Since the cloning of OCTN1 and OCTN2 in the late 1990s, many studies have investigated their function, expression, and regulation due to their involvement in human pathologies, such as primary carnitine deficiency, inflammatory-based diseases, and cancer. Even in the absence of a 3D structure, several aspects of the physiopathological role of the two proteins have been clarified, leaving underexplored the transcriptional regulation of the two genes. The promoter/enhancer region of the two genes has been investigated, and the putative transcription factors involved in the regulation have been predicted by bioinformatics. An antisense lncRNA, MIR3936HG, with an unknown function, probably involved in *OCTN* regulation, has been described for the first time. Searching for the *OCTN1* missense mutations in the protein homology model highlights the proximity of some of them to the binding site(s). Site-directed mutagenesis studies on selected mutants will unveil their role in the translocation pathway in physiological and pathological contexts such as cancer.

## Figures and Tables

**Table 1:** Transcription factors of the OCTN1 promoter/enhancer region predicted by JASPAR.

Transcription Factor	JASPAR Score	Genomic Position	Binding Site Size
ZNF354A	637	chr5:132292671-132292690	20
ZSCAN16	670 602	chr5:132292976-132292993 chr5:132296737-132296754	18
ZNF75D	607	chr5:132293083-132293094	12
ZKSCAN3	608	chr5:132293266-132293279	14
PRDM9	657	chr5:132293697-132293716	20
ZNF454	636	chr5:132294069-132294085	17
ZNF460	668 621 745	chr5:132294326-132294341 chr5:132297073-132297088 chr5:132297208-132297223	16
ZNF816	605	chr5:132294499-132294513	15
ZNF281	602	chr5:132295107-132295116	10
ZNF148	602	chr5:132295107-132295116	10
EWSR1-FLI1	628 616 728	chr5:132296985-132297002 chr5:132297372-132297389 chr5:132297376-132297393	18
Nr1h3::Rxra	603	chr5:132297093-132297108	16
FOXD3	602	chr5:132297318-132297331	14

**Table 2:** *OCTN1* transcripts.

Gene Symbol	Database	Transcript	Length (nt)
*SLC22A4*	NCBI/Gene	NM_003059.3	2234
*SLC22A4*	NCBI/Gene	XM_006714675.5	2130
*SLC22A4*	NCBI/Gene	XM_011543589.3	1958
*SLC22A4*	NCBI/Gene	XM_047417594.1	1788
*SLC22A4*	NCBI/Gene	XM_017009776.2	1821
*SLC22A4*	Ensembl	ENST00000491257.1	564
*SLC22A4*	Ensembl	ENST00000425923.1	463

**Table 3:** Transcription factors of the *OCTN2* promoter/enhancer region predicted by JASPAR.

Transcription Factor	JASPAR Score	Genomic Position	Binding Site Size
ZNF460	776	chr5:132368308-132368323	16
ZBED4	602	chr5:132369676-132369685	10
ZNF93	633	chr5:132370212-132370225	14
PATZ1	614	chr5:132370470-132370480	11
ZNF281	602	chr5:132370471-132370480	10
ZNF148	602	chr5:132370471-132370480	10
Nr2F6	600	chr5:132371186-132371200	15
PPARA::RXRA	717	chr5:132372636-132372652	17

**Table 4:** *OCTN2* transcripts.

Gene Symbol	Database	Transcript	Trasncript Length (nt)	Protein Length (aa)
*SLC22A5*	NCBI/Gene	NM_001308122.2	3349	581
*SLC22A5*	NCBI/Gene	NM_003060.4	3277	557
*SLC22A5*	NCBI/Gene	XM_017009778.3	1570	381
*SLC22A5*	NCBI/Gene	XM_047417595.1	1788	374
*SLC22A5*	NCBI/Gene	XM_047417596.1	8198	353
*SLC22A5*	NCBI/Gene	XM_011543590.3	2495	351
*SLC22A5*	NCBI/Gene	XM_047417597.1	1295	308
*SLC22A5*	NCBI/Gene	XM_047417598.1	1289	306
*SLC22A5*	Ensembl	ENST00000693308.1	3146	573
*SLC22A5*	Ensembl	ENST00000692413.1	3080	551
*SLC22A5*	Ensembl	ENST00000689271.1	2945	506
*SLC22A5*	Ensembl	ENST00000415928.6	1873	431

## References

[1] Ferrada E., Superti-Furga G. (2022). A structure and evolutionary-based classification of solute carriers. iScience.

[2] Dobson L., Remenyi I., Tusnady G.E. (2015). The human transmembrane proteome. Biol. Direct.

[3] Koepsell H. (2020). Organic Cation Transporters in Health and Disease. Pharmacol. Rev..

[4] Suo Y., Wright N.J., Guterres H., Fedor J.G., Butay K.J., Borgnia M.J., Im W., Lee S.Y. (2023). Molecular basis of polyspecific drug and xenobiotic recognition by OCT1 and OCT2. Nat. Struct. Mol. Biol..

[5] Khanppnavar B., Maier J., Herborg F., Gradisch R., Lazzarin E., Luethi D., Yang J.W., Qi C., Holy M., Jantsch K. (2022). Structural basis of organic cation transporter-3 inhibition. Nat. Commun..

[6] Zeng Y.C., Sobti M., Quinn A., Smith N.J., Brown S.H.J., Vandenberg J.I., Ryan R.M., O’Mara M.L., Stewart A.G. (2023). Structural basis of promiscuous substrate transport by Organic Cation Transporter 1. Nat. Commun..

[7] Zhang S., Zhu A., Kong F., Chen J., Lan B., He G., Gao K., Cheng L., Sun X., Yan C. (2024). Structural insights into human organic cation transporter 1 transport and inhibition. Cell Discov..

[8] Parker J.L., Kato T., Kuteyi G., Sitsel O., Newstead S. (2023). Molecular basis for selective uptake and elimination of organic anions in the kidney by OAT1. Nat. Struct. Mol. Biol..

[9] Pochini L., Barone F., Console L., Brunocilla C., Galluccio M., Scalise M., Indiveri C. (2024). OCTN1 (SLC22A4) displays two different transport pathways for organic cations or zwitterions. Biochim. Biophys. Acta Biomembr..

[10] Tamai I. (2013). Pharmacological and pathophysiological roles of carnitine/organic cation transporters (OCTNs: SLC22A4, SLC22A5 and Slc22a21). Biopharm. Drug Dispos..

[11] Enomoto A., Wempe M.F., Tsuchida H., Shin H.J., Cha S.H., Anzai N., Goto A., Sakamoto A., Niwa T., Kanai Y. (2002). Molecular identification of a novel carnitine transporter specific to human testis. Insights into the mechanism of carnitine recognition. J. Biol. Chem..

[12] Galluccio M., Mazza T., Scalise M., Sarubbi M.C., Indiveri C. (2022). Bacterial over-expression of functionally active human CT2 (SLC22A16) carnitine transporter. Mol. Biol. Rep..

[13] Koepsell H., Endou H. (2004). The SLC22 drug transporter family. Pflügers Arch..

[14] Pochini L., Galluccio M., Console L., Scalise M., Eberini I., Indiveri C. (2024). Inflammation and Organic Cation Transporters Novel (OCTNs). Biomolecules.

[15] Pochini L., Galluccio M., Scalise M., Console L., Pappacoda G., Indiveri C. (2022). OCTN1: A Widely Studied but Still Enigmatic Organic Cation Transporter Linked to Human Pathology and Drug Interactions. Int. J. Mol. Sci..

[16] Yamamoto-Furusho J.K., Mendivil E.J., Villeda-Ramirez M.A., Fonseca-Camarillo G., Barreto-Zuniga R. (2011). Gene expression of carnitine organic cation transporters 1 and 2 (OCTN) is downregulated in patients with ulcerative colitis. Inflamm. Bowel Dis..

[17] Rioux J.D., Silverberg M.S., Daly M.J., Steinhart A.H., McLeod R.S., Griffiths A.M., Green T., Brettin T.S., Stone V., Bull S.B. (2000). Genomewide search in Canadian families with inflammatory bowel disease reveals two novel susceptibility loci. Am. J. Hum. Genet..

[18] Park H.J., Jung E.S., Kong K.A., Park E.M., Cheon J.H., Choi J.H. (2016). Identification of OCTN2 variants and their association with phenotypes of Crohn’s disease in a Korean population. Sci. Rep..

[19] Friedman J.R., Richbart S.D., Merritt J.C., Brown K.C., Nolan N.A., Akers A.T., Lau J.K., Robateau Z.R., Miles S.L., Dasgupta P. (2019). Acetylcholine signaling system in progression of lung cancers. Pharmacol. Ther..

[20] Shibbani K., Fahed A.C., Al-Shaar L., Arabi M., Nemer G., Bitar F., Majdalani M. (2014). Primary carnitine deficiency: Novel mutations and insights into the cardiac phenotype. Clin. Genet..

[21] Nezu J., Tamai I., Oku A., Ohashi R., Yabuuchi H., Hashimoto N., Nikaido H., Sai Y., Koizumi A., Shoji Y. (1999). Primary systemic carnitine deficiency is caused by mutations in a gene encoding sodium ion-dependent carnitine transporter. Nat. Genet..

[22] Stanley C.A. (2004). Carnitine deficiency disorders in children. Ann. N. Y. Acad. Sci..

[23] Frigeni M., Balakrishnan B., Yin X., Calderon F.R.O., Mao R., Pasquali M., Longo N. (2017). Functional and molecular studies in primary carnitine deficiency. Hum. Mutat..

[24] Tang N.L., Ganapathy V., Wu X., Hui J., Seth P., Yuen P.M., Wanders R.J., Fok T.F., Hjelm N.M. (1999). Mutations of OCTN2, an organic cation/carnitine transporter, lead to deficient cellular carnitine uptake in primary carnitine deficiency. Hum. Mol. Genet..

[25] Tokuhiro S., Yamada R., Chang X., Suzuki A., Kochi Y., Sawada T., Suzuki M., Nagasaki M., Ohtsuki M., Ono M. (2003). An intronic SNP in a RUNX1 binding site of SLC22A4, encoding an organic cation transporter, is associated with rheumatoid arthritis. Nat. Genet..

[26] Rioux J.D., Daly M.J., Silverberg M.S., Lindblad K., Steinhart H., Cohen Z., Delmonte T., Kocher K., Miller K., Guschwan S. (2001). Genetic variation in the 5q31 cytokine gene cluster confers susceptibility to Crohn disease. Nat. Genet..

[27] Peltekova V.D., Wintle R.F., Rubin L.A., Amos C.I., Huang Q., Gu X., Newman B., Van Oene M., Cescon D., Greenberg G. (2004). Functional variants of OCTN cation transporter genes are associated with Crohn disease. Nat. Genet..

[28] Wang J., Wang X., Yang H., Wu D., Wang L., Qian J. (2011). Contribution of the IBD5 locus to inflammatory bowel disease: A meta-analysis. Hum. Genet..

[29] Pochini L., Scalise M., Galluccio M., Pani G., Siminovitch K.A., Indiveri C. (2012). The human OCTN1 (SLC22A4) reconstituted in liposomes catalyzes acetylcholine transport which is defective in the mutant L503F associated to the Crohn’s disease. Biochim. Biophys. Acta.

[30] Wagner J., Sim W.H., Ellis J.A., Ong E.K., Catto-Smith A.G., Cameron D.J., Bishop R.F., Kirkwood C.D. (2010). Interaction of Crohn’s disease susceptibility genes in an Australian paediatric cohort. PLoS ONE.

[31] Santiago J.L., Martinez A., de la Calle H., Fernandez-Arquero M., Figueredo M.A., de la Concha E.G., Urcelay E. (2006). Evidence for the association of the SLC22A4 and SLC22A5 genes with type 1 diabetes: A case control study. BMC Med. Genet..

[32] Klaassen C.D., Aleksunes L.M. (2010). Xenobiotic, bile acid, and cholesterol transporters: Function and regulation. Pharmacol. Rev..

[33] Pochini L., Galluccio M., Scalise M., Console L., Indiveri C. (2019). OCTN: A Small Transporter Subfamily with Great Relevance to Human Pathophysiology, Drug Discovery, and Diagnostics. SLAS Discov..

[34] Rattray N.J.W., Trivedi D.K., Xu Y., Chandola T., Johnson C.H., Marshall A.D., Mekli K., Rattray Z., Tampubolon G., Vanhoutte B. (2019). Metabolic dysregulation in vitamin E and carnitine shuttle energy mechanisms associate with human frailty. Nat. Commun..

[35] Kim E., Han D.J., Kim B.H., Yoo J., Kim H.J., Wu H.G., Kim K.S., Kim H.S., Han I., Moon K.C. (2023). Whole-Genome Sequencing Reveals Mutational Signatures Related to Radiation-Induced Sarcomas and DNA-Damage-Repair Pathways. Mod. Pathol..

[36] Maeda T., Hirayama M., Kobayashi D., Miyazawa K., Tamai I. (2007). Mechanism of the regulation of organic cation/carnitine transporter 1 (SLC22A4) by rheumatoid arthritis-associated transcriptional factor RUNX1 and inflammatory cytokines. Drug Metab. Dispos..

[37] Tahara H., Yee S.W., Urban T.J., Hesselson S., Castro R.A., Kawamoto M., Stryke D., Johns S.J., Ferrin T.E., Kwok P.Y. (2009). Functional genetic variation in the basal promoter of the organic cation/carnitine transporters OCTN1 (SLC22A4) and OCTN2 (SLC22A5). J. Pharmacol. Exp. Ther..

[38] Jung E.S., Park H.J., Kong K.A., Choi J.H., Cheon J.H. (2017). Association study between OCTN1 functional haplotypes and Crohn’s disease in a Korean population. Korean J. Physiol. Pharmacol..

[39] Angelini S., Soverini S., Ravegnini G., Barnett M., Turrini E., Thornquist M., Pane F., Hughes T.P., White D.L., Radich J. (2013). Association between imatinib transporters and metabolizing enzymes genotype and response in newly diagnosed chronic myeloid leukemia patients receiving imatinib therapy. Haematologica.

[40] Machova Polakova K., Albeer A., Polivkova V., Krutska M., Vlcanova K., Curik N., Fabarius A., Klamova H., Spiess B., Waller C.F. (2024). The SNP rs460089 in the gene promoter of the drug transporter OCTN1 has prognostic value for treatment-free remission in chronic myeloid leukemia patients treated with imatinib. Leukemia.

[41] Fishilevich S., Nudel R., Rappaport N., Hadar R., Plaschkes I., Iny Stein T., Rosen N., Kohn A., Twik M., Safran M. (2017). GeneHancer: Genome-wide integration of enhancers and target genes in GeneCards. Database.

[42] Rauluseviciute I., Riudavets-Puig R., Blanc-Mathieu R., Castro-Mondragon J.A., Ferenc K., Kumar V., Lemma R.B., Lucas J., Cheneby J., Baranasic D. (2024). JASPAR 2024: 20th anniversary of the open-access database of transcription factor binding profiles. Nucleic Acids Res..

[43] Tamai I., Yabuuchi H., Nezu J., Sai Y., Oku A., Shimane M., Tsuji A. (1997). Cloning and characterization of a novel human pH-dependent organic cation transporter, OCTN1. FEBS Lett..

[44] Liao B., Wang J., Yuan Y., Luo H., Ouyang X. (2024). Biological roles of SLC16A1-AS1 lncRNA and its clinical impacts in tumors. Cancer Cell Int..

[45] Magdy T., Jouni M., Kuo H.H., Weddle C.J., Lyra-Leite D., Fonoudi H., Romero-Tejeda M., Gharib M., Javed H., Fajardo G. (2022). Identification of Drug Transporter Genomic Variants and Inhibitors That Protect Against Doxorubicin-Induced Cardiotoxicity. Circulation.

[46] Kobayashi D., Aizawa S., Maeda T., Tsuboi I., Yabuuchi H., Nezu J., Tsuji A., Tamai I. (2004). Expression of organic cation transporter OCTN1 in hematopoietic cells during erythroid differentiation. Exp. Hematol..

[47] Longo N. (2016). Primary Carnitine Deficiency and Newborn Screening for Disorders of the Carnitine Cycle. Ann. Nutr. Metab..

[48] Koleske M.L., McInnes G., Brown J.E.H., Thomas N., Hutchinson K., Chin M.Y., Koehl A., Arkin M.R., Schlessinger A., Gallagher R.C. (2022). Functional genomics of OCTN2 variants informs protein-specific variant effect predictor for Carnitine Transporter Deficiency. Proc. Natl. Acad. Sci. USA.

[49] Tamai I., Ohashi R., Nezu J., Yabuuchi H., Oku A., Shimane M., Sai Y., Tsuji A. (1998). Molecular and functional identification of sodium ion-dependent, high affinity human carnitine transporter OCTN2. J. Biol. Chem..

[50] Maekawa S., Mori D., Nishiya T., Takikawa O., Horinouchi T., Nishimoto A., Kajita E., Miwa S. (2007). OCTN2VT, a splice variant of OCTN2, does not transport carnitine because of the retention in the endoplasmic reticulum caused by insertion of 24 amino acids in the first extracellular loop of OCTN2. Biochim. Biophys. Acta.

[51] Pochini L., Scalise M., Galluccio M., Indiveri C. (2013). OCTN cation transporters in health and disease: Role as drug targets and assay development. J. Biomol. Screen..

[52] Ganapathy M.E., Huang W., Rajan D.P., Carter A.L., Sugawara M., Iseki K., Leibach F.H., Ganapathy V. (2000). beta-lactam antibiotics as substrates for OCTN2, an organic cation/carnitine transporter. J. Biol. Chem..

[53] Ohnishi S., Okamura N., Sakamoto S., Hasegawa H., Norikura R., Kanaoka E., Takahashi K., Horie K., Sakamoto K., Baba T. (2008). Role of Na+/L-carnitine transporter (OCTN2) in renal handling of pivaloylcarnitine and valproylcarnitine formed during pivalic acid-containing prodrugs and valproic acid treatment. Drug Metab. Pharmacokinet..

[54] Nakahara S., Arimura Y., Saito K., Goto A., Motoya S., Shinomura Y., Miyamoto A., Imai K. (2008). Association of SLC22A4/5 polymorphisms with steroid responsiveness of inflammatory bowel disease in Japan. Dis. Colon Rectum.

[55] Heintzman N.D., Stuart R.K., Hon G., Fu Y., Ching C.W., Hawkins R.D., Barrera L.O., Van Calcar S., Qu C., Ching K.A. (2007). Distinct and predictive chromatin signatures of transcriptional promoters and enhancers in the human genome. Nat. Genet..

[56] Qu Q., Qu J., Zhan M., Wu L.X., Zhang Y.W., Lou X.Y., Fu L.J., Zhou H.H. (2013). Different involvement of promoter methylation in the expression of organic cation/carnitine transporter 2 (OCTN2) in cancer cell lines. PLoS ONE.

[57] He R.Z., Luo D.X., Mo Y.Y. (2019). Emerging roles of lncRNAs in the post-transcriptional regulation in cancer. Genes Dis..

[58] Liang X.H., Shen W., Sun H., Migawa M.T., Vickers T.A., Crooke S.T. (2016). Translation efficiency of mRNAs is increased by antisense oligonucleotides targeting upstream open reading frames. Nat. Biotechnol..

[59] Zhou S., Shu Y. (2022). Transcriptional Regulation of Solute Carrier (SLC) Drug Transporters. Drug Metab. Dispos..

[60] Oscarson M., Zanger U.M., Rifki O.F., Klein K., Eichelbaum M., Meyer U.A. (2006). Transcriptional profiling of genes induced in the livers of patients treated with carbamazepine. Clin. Pharmacol. Ther..

[61] Paul H.S., Adibi S.A. (1979). Paradoxical effects of clofibrate on liver and muscle metabolism in rats. Induction of myotonia and alteration of fatty acid and glucose oxidation. J. Clin. Investig..

[62] Luci S., Geissler S., Konig B., Koch A., Stangl G.I., Hirche F., Eder K. (2006). PPARalpha agonists up-regulate organic cation transporters in rat liver cells. Biochem. Biophys. Res. Commun..

[63] Schoonjans K., Peinado-Onsurbe J., Lefebvre A.M., Heyman R.A., Briggs M., Deeb S., Staels B., Auwerx J. (1996). PPARalpha and PPARgamma activators direct a distinct tissue-specific transcriptional response via a PPRE in the lipoprotein lipase gene. EMBO J..

[64] Ringseis R., Posel S., Hirche F., Eder K. (2007). Treatment with pharmacological peroxisome proliferator-activated receptor alpha agonist clofibrate causes upregulation of organic cation transporter 2 in liver and small intestine of rats. Pharmacol. Res..

[65] van Vlies N., Wanders R.J., Vaz F.M. (2006). Measurement of carnitine biosynthesis enzyme activities by tandem mass spectrometry: Differences between the mouse and the rat. Anal. Biochem..

[66] Ringseis R., Luci S., Spielmann J., Kluge H., Fischer M., Geissler S., Wen G., Hirche F., Eder K. (2008). Clofibrate treatment up-regulates novel organic cation transporter (OCTN)-2 in tissues of pigs as a model of non-proliferating species. Eur. J. Pharmacol..

[67] D’Argenio G., Petillo O., Margarucci S., Torpedine A., Calarco A., Koverech A., Boccia A., Paolella G., Peluso G. (2010). Colon OCTN2 gene expression is up-regulated by peroxisome proliferator-activated receptor gamma in humans and mice and contributes to local and systemic carnitine homeostasis. J. Biol. Chem..

[68] Grube M., Meyer Zu Schwabedissen H., Draber K., Prager D., Moritz K.U., Linnemann K., Fusch C., Jedlitschky G., Kroemer H.K. (2005). Expression, localization, and function of the carnitine transporter octn2 (slc22a5) in human placenta. Drug Metab. Dispos..

[69] Huang F.D., Kung F.L., Tseng Y.C., Chen M.R., Chan H.S., Lin C.J. (2009). Regulation of protein expression and function of octn2 in forskolin-induced syncytialization in BeWo Cells. Placenta.

[70] Chang T.T., Shyu M.K., Huang M.C., Hsu C.C., Yeh S.Y., Chen M.R., Lin C.J. (2011). Hypoxia-mediated down-regulation of OCTN2 and PPARalpha expression in human placentas and in BeWo cells. Mol. Pharm..

[71] Eder K., Ringseis R. (2010). The role of peroxisome proliferator-activated receptor alpha in transcriptional regulation of novel organic cation transporters. Eur. J. Pharmacol..

[72] Fink M.A., Paland H., Herzog S., Grube M., Vogelgesang S., Weitmann K., Bialke A., Hoffmann W., Rauch B.H., Schroeder H.W.S. (2019). L-Carnitine-Mediated Tumor Cell Protection and Poor Patient Survival Associated with OCTN2 Overexpression in Glioblastoma Multiforme. Clin. Cancer Res..

[73] Elsnerova K., Mohelnikova-Duchonova B., Cerovska E., Ehrlichova M., Gut I., Rob L., Skapa P., Hruda M., Bartakova A., Bouda J. (2016). Gene expression of membrane transporters: Importance for prognosis and progression of ovarian carcinoma. Oncol. Rep..

[74] Scalise M., Galluccio M., Accardi R., Cornet I., Tommasino M., Indiveri C. (2012). Human OCTN2 (SLC22A5) is down-regulated in virus- and nonvirus-mediated cancer. Cell Biochem. Funct..

[75] Wang C., Uray I.P., Mazumdar A., Mayer J.A., Brown P.H. (2012). SLC22A5/OCTN2 expression in breast cancer is induced by estrogen via a novel intronic estrogen-response element (ERE). Breast Cancer Res. Treat..

[76] Papierniak-Wygladala A., Lamch W., Jurewicz E., Nalecz K.A. (2023). The activity and surface presence of organic cation/carnitine transporter OCTN2 (SLC22A5) in breast cancer cells depends on AKT kinase. Arch. Biochem. Biophys..

[77] Jing Z., Okubo H., Morishige J.I., Xu P., Hasan N., Nagata N., Ando H. (2022). Lenvatinib causes reduced expression of carnitine/organic cation transporter 2 and carnitine deficiency in the skeletal muscle of rats. Toxicol. Lett..

[78] Juraszek B., Nalecz K.A. (2019). SLC22A5 (OCTN2) Carnitine Transporter-Indispensable for Cell Metabolism, a Jekyll and Hyde of Human Cancer. Molecules.

